# The variants of polymyxin susceptibility in different species of genus *Aeromonas*

**DOI:** 10.3389/fmicb.2022.1030564

**Published:** 2022-10-25

**Authors:** Linna Xu, Junfeng Fan, Hao Fu, Yuyi Yang, Qixia Luo, Fen Wan

**Affiliations:** ^1^School of Laboratory Medicine and Biotechnology, Hangzhou Medical College, Hangzhou, China; ^2^State Key Laboratory for Diagnosis and Treatment of Infectious Diseases, Collaborative Innovation Center for Diagnosis and Treatment of Infectious Diseases, The First Affiliated Hospital of Medical School, College of Medicine, Zhejiang University, Hangzhou, China; ^3^Wuhan Botanical Garden, Chinese Academy of Sciences, Wuhan, China

**Keywords:** aquatic environment, polymyxin resistance, *mcr*, MlaF, *Aeromonas*

## Abstract

The aquatic environment is an important medium for the accumulation and dissemination of antibiotic-resistant bacteria as it is often closely related to human activities. Previous studies paid little attention to the prevalence and mechanism of polymyxin-resistant bacteria in the aquatic environment. As a Gram-negative opportunistic pathogen widely distributed in aquatic ecosystems, the antibiotic-resistant profile of *Aeromonas* spp. deserves much attention. In this study, we identified 61 *Aeromonas* spp. isolates from water samples in the section of the Yangtze River. The total polymyxin B (PMB) resistance rate of these strains was 49.18% (30/61), showing a high level of polymyxin resistance in *Aeromonas* spp. The MIC_50_ and MIC_90_ for PMB exhibited a significant discrepancy among different species (*p* < 0.001). The MIC_50_ and MIC_90_ for PMB in the *Aeromonas hydrophila* were 128 mg/L and above 128 mg/L while in *Aeromonas caviae* and *Aeromonas veronii*, the MIC_50_ and MIC_90_ value were both 2 mg/L. Only two *A*. *veronii* strains (MIC = 2 mg/L) and one *A*. *caviae* strain (MIC = 0.5 mg/L) were identified as carrying mobilized polymyxin resistant gene *mcr-3*.*42*, and *mcr-3*.*16*. All *mcr* genes were located in the chromosome. This is the first report that the downstream region of *mcr*-3.42 was the truncated *mcr-3*-like gene separated by the insertion sequences of IS*As20* (1,674 bp) and IS*As2* (1,084 bp). Analysis of epidemiology of *mcr*-positive *Aeromonas* genomes from GenBank database showed that the genus *Aeromonas* and the aquatic environment might be the potential container and reservoir of *mcr-3*. By the whole-genome sequencing and qRT-PCR, we inferred that the sequence differences in the AAA domain of MlaF protein and its expression level among these three species might be involved in the development of polymyxin resistance. Our study provided evidences of the possible mechanism for the variety of polymyxin susceptibility in different species of the genus *Aeromonas* and a theoretical basis for the surveillance of the aquatic environment.

## Introduction

Antimicrobial resistance (AMR) has been a major challenge to global public health with the use of antibiotics over the decades. Antimicrobial resistance genes (ARGs) are considered as environmental pollutants as the result of the continuous release of residual antibiotics by human activities into the environment such as hospitals, livestock, and sewage treatment plant, which increased occurrence of antimicrobial resistant bacteria (ARB) (Pruden et al., 2006; Zurfluh et al., 2017). Moreover, the aquatic environment itself can serve as the sites of the spreading of AMR and the reservoir of ARGs, like *qnrA*, *qnrB*, *qnrS*, *mef*, and so on (Pei et al., 2006; Aedo et al., 2014; Nonaka et al., 2015). There are a large number of bacteria (> 100 million/liter of seawater) in the aquatic environment, from which bacteria such as *Aeromonas* spp., *Vibrio*, and *Pseudomonas* sp. are often isolated (Noonburg, 2005). Especially, *Aeromonas* spp., consisting of 30 species, is widely distributed in various kinds of natural water ecosystems (Chen et al., 2021). *Aeromonas* spp. are gram-negative, rod-shaped aerobes belonging to the *Aeromonadaceae* family. Among the members of *Aeromonas* spp., *Aeromonas hydrophila*, *Aeromonas veronii*, and *Aeromonas caviae* are the common zoonotic pathogens both for human and aquatic animals. In recent years, *Aeromonas* species have become the third most common enteric bacterial pathogens, with a clinical isolation rate of 56.73% (Yuwono et al., 2021). The antimicrobial susceptibility of *Aeromonas* has been reported in many studies, showing that *Aeromonas* were resistant to ampicillin, penicillin, and cephalosporins, tetracycline (Scarano et al., 2018; Jia et al., 2020; Sun et al., 2021).

Polymyxins, including polymyxin B (PMB) and polymyxin E (colistin), are a group of cationic polypeptides that disrupts membrane integrity by replacing cations like Mg^2+^ and Ca^2+^ in the outer membrane, which leads to the cell lysis. Due to the emergence of bacteria resistant to the most common-use antibiotic and the shortage of new antimicrobial agents to resist them, polymyxins have become the last line of defense against multiple drug-resistant gram-negative bacteria infections (Falagas and Rafailidis, 2008; Trimble et al., 2016). The most common mechanism of colistin resistance is the chromosomal mutations in genes involved in the synthesis and modification of lipid A of lipopolysaccharide (LPS). These genes include the *arnBCADTEF* operon which is regulated by the two-component system (TCS) PhoP/Q, PmrA/B, ParR/S, CprR/S, and ColR/S (Olaitan et al., 2014). In addition to that, the horizontal transfer of the plasmid-carrying gene *mcr* encoding phosphoethanolamine (PEtN) transferase which modifies the lipid A by addition of phosphoethanolamine has become another important cause of colistin resistance. Since the first plasmid-mediated colistin resistance gene *mcr-1* was identified in 2015, a total of 10 *mcr* gene variants (*mcr-1* to *mcr-10*) have been reported, mainly among the Enterobacterales (Hussein et al., 2021).

In *Aeromonas* species, *mcr-2*, *mcr-3*, *mcr-5*, and *mcr-7* have been reported, especially the variants of *mcr-3*, which were widely detected and frequently disseminated between Enterobacteriaceae and *Aeromonas* (Ma et al., 2018; Sun et al., 2018; Ragupathi et al., 2020; Wang et al., 2021). Beyond that, the *mcr*-positive *Aeromonas* strains showed the discrepancy of polymyxin-resistant phenotype, manifesting as a wide span of and the minimum inhibitory concentration (MIC) of polymyxin ranging from <0.5 mg/L to >128 mg/L. But the exact mechanism of polymyxin resistance in *Aeromonas* and reasons for the differences of polymyxin susceptibility in different *Aeromonas* species remains poorly understood and less attention has been paid to aquatic environments (Ling et al., 2017; Tuo et al., 2018; Ragupathi et al., 2020).

Here, we determined the polymyxin susceptibility in different *Aeromonas* species isolated from the section of the Yangtze River, investigated the prevalence of the *mcr* gene, and explored the connections between gene expression levels in the TCS involved in the synthesis and modification of lipid A and the polymyxin resistance. A total of 61 *Aeromonas* strains were detected and 30 strains showed polymyxin resistance, showing a high level of polymyxin resistance rate of 49.18% (30/61). Among different species in the genus *Aeromonas*, the polymyxin resistance rate showed a distinct discrepancy (*p* < 0.001). Two *A*. *veronii* strains were identified as *mcr-3*.*42* positive strains, and one *A*. *caviae* strain was identified as *mcr-3*.*16* while the rest strains remained negative of *mcr-1* to *mcr-10*. We inferred that the existence of *arnBCADTEF* and mutations in MlaF protein among different *Aeromonas* species could affect the function of phospholipid transportation and contribute to the resistance to polymyxins.

## Materials and methods

### Bacterial isolation and identification

To investigate the dissemination of *mcr* in the genus *Aeromonas* from the aquatic environment, 16 water samples were collected in sterile 1-L bottles from A section of the Yangtze River. All samples were stored at 4°C after collection and analyzed within 12 h. Luria-Bertani (LB), Mueller-Hinton (MH), and Salmonella Shigella (SS) culture mediums were used to perform the culture and isolation of bacteria. The whole isolates were screened for the presence of *mcr-1* to *mcr-10* by colony PCR (Woodman et al., 2016). The positive PCR products were verified by Sanger sequencing. Primer pairs used in this study were listed in Supplementary Table S1. Matrix-assisted laser desorption ionization-time of flight (MALDI-TOF) MS identification and 16S rRNA sequencing were adopted for bacterial identification.

### Antimicrobial susceptibility testing and microbiological assessment

Antimicrobial susceptibility testing (AST) was conducted by the broth microdilution method and agar dilution method. Testing was performed according to Clinical and Laboratory Standards Institute guidelines (CLSI) (2021). The minimum inhibitory concentration (MIC) of PMB (Sigma, Shanghai, China), tigecycline (TGC), and tetracycline (TCY) (Solarbio, Shanghai, China) were determined by the broth microdilution method, and the MIC of common drugs for clinical therapy including amikacin, cefepime, ciprofloxacin, meropenem, aztreonam et al. (Solarbio, Shanghai, China) were determined by agar dilution method. The breakpoint of TGC was interpreted according to the guidelines of the European Committee on Antimicrobial Susceptibility Testing (EUCAST) version 10.0.^1^ The other antimicrobial agents were interpreted according to the guidelines of the CLSI M100 (2021 version, 31st edition) and M45-A2 (2015 version, 3rd edition) documents. *Escherichia coli* ATCC 25922 was used as the quality control strain for antimicrobial susceptibility testing.

### Conjugation experiment and S1 nuclease pulsed-field gel electrophoresis analysis

The conjugation experiment was carried out to investigate the transferability of *mcr* gene. Procedures were performed according to the method described previously (Hadjadj et al., 2019). Sodium azide-resistant *E*. *coli* J53 (MIC of 200 mg/L) was used as recipient strain. The transconjugants were selected on the medium containing azide at 200 mg/L and PMB, at 1 mg/L was consistent with the MIC of the donor. Resultant colonies were screened for the presence of *mcr* by PCR.

S1-PFGE analysis of *mcr-*positive isolates was used to estimate the sizes of *mcr*-positive plasmids. *Xba*I-restricted DNA of *Salmonella enterica* serovar Braenderup H9812 was used as a DNA marker. Shortly, agarose plugs were made using the colonies in the medium of a single colony inoculated and digested with S1 nuclease (TaKaRa, Dalian, China). The DNA was separated using the CHEF-MAPPER PFGE system (Bio-Rad) under the following conditions: 14°C, 6 V/cm, and a 120° pulse angle for 16 h, with the initial and final pulses conducted for 2.16 s and 63.8 s, respectively. Then the dyed gel was visualized with the imaging system.

### DNA extraction, whole-genome sequencing, and genomic analyses

Genomic DNA of *mcr*-positive isolates were extracted using Gentra Puregene Yeast/Bact. Kit (Qiagen, CA, United States), according to the manufacturer’s instructions. Illumina Hiseq PE150 platform was used for the whole-genome sequencing (WGS). After read quality control, the reads from Illumina Hiseq platform were assembled using SPAdes 3.10.18 which is based on De-Bruijn algorithm and takes the appropriate k-mer values. Genetic predictions of assembly results were using the web platforms of the National Center for Biotechnological Information (NCBI) and ORFfinder.^2^ The sequences were annotated using the RAST annotation server^3^ and prokka 1.14.6.^4^ Assembled contigs were analyzed *via* the Center for Genomic Epidemiology website^5^ to screen for the presence of acquired AMR genes, plasmid incompatibility types, and Multi Locus Sequence Typing (MLST). Linear comparisons of *mcr* were generated using Easyfig. 2. Multiple alignments of nucleotide sequences were performed by using The European Bioinformatics Institute (EMBL-EBI) tools.^6^ To investigate the distribution of *mcr*-positive strains of the genus *Aeromonas*, we retrieved the isolates in sequences deposited in GenBank by Genomes Browser,^7^ (accessed 26 April 2022) and Isolates Browser^8^ (accessed 26 April 2022).

### Reverse transcription-quantitative PCR

Total RNA was extracted from mid-long phase (OD_600_ ~ 0.6) bacterial cultures using the Qiagen RNeasy Mini kit (QIAGEN, Alameda, United States), according to the manufacturer’s instructions. Complementary DNA (cDNA) was generated from total RNA using the RT Premix. Supplementary Table S2 shows the sequences of transcript-specific primers used for qPCR. All samples were performed in triplicate, and the data were normalized to 16S rRNA levels and analyzed using the 2^−ΔΔCT^ method to calculate the fold-change relative to the control. The difference between groups was analyzed by Welch’s *t*-test.

### Amino acids analysis and protein structure prediction

Multiple alignments of amino acid sequences were performed by using The European Bioinformatics Institute (EMBL-EBI) tools^9^ and Jalview 2.11.1.4 was used to perform the multiple sequence alignment editing, visualization, and analysis. PROVEAN Protein tools^10^ were calculated to evaluate whether amino acid alterations in PhoP/PhoQ, OmpR/EnvZ, and MlaF affected biological function. Moreover, the protein domains of PhoP/PhoQ, OmpR/EnvZ, and MlaF were subjected to SMART analysis.^11^ The secondary structure and complex structure of MlaF protein were predicted by PSIPRED^12^ and SWISS-MODEL,^13^ respectively. The images of biomolecular structures of MlaF protein were generated by PyMoL (Seeliger and de Groot, 2010).

### Nucleotide sequence accession numbers

The whole-genome nucleotide sequences of 15 *Aeromonas* strains have been submitted to DDBJ/ENA/GenBank under the accession numbers JANKLR000000000-JANKLZ000000000, JANKMA000000000-JANKME000000000, and JANPYL000000000 and were in a processing queue (BioProject: PRJNA864564). The accession numbers were listed in Table 1.

**Table 1:** Characteristics of *mcr*-3 and *mcr*-negative *Aeromonas* strains isolated in this study.

Isolate	Species	PMB MIC (mg/L)	Isolate source	*mcr*	Accession
S461	*A*. *hydrophila*	>128	Yangtze River	*mcr*-negative	JANPYL000000000
S541	*A*. *hydrophila*	>128	Yangtze River	*mcr*-negative	JANKLR000000000
M894	*A*. *hydrophila*	>128	Yangtze River	*mcr*-negative	JANKLS000000000
L652	*A*. *hydrophila*	>128	Yangtze River	*mcr*-negative	JANKLT000000000
M621	*A*. *caviae*	1	Yangtze River	*mcr*-negative	JANKLU000000000
L7105	*A*. *caviae*	2	Yangtze River	*mcr*-negative	JANKLV000000000
L965	*A*. *caviae*	1	Yangtze River	*mcr*-negative	JANKLW000000000
L215	*A*. *caviae*	1	Yangtze River	*mcr*-negative	JANKLX000000000
S5183	*A*. *veronii*	2	Yangtze River	*mcr*-negative	JANKLY000000000
M194	*A*. *veronii*	2	Yangtze River	*mcr*-negative	JANKLZ000000000
L975	*A*. *veronii*	1	Yangtze River	*mcr*-negative	JANKMA000000000
L924	*A*. *veronii*	2	Yangtze River	*mcr*-negative	JANKMB000000000
S611	*A*. caviae	0.5	Yangtze River	*mcr*-3	JANKMC000000000
M694	*A*. *veronii*	2	Yangtze River	*mcr*-3	JANKMD000000000
M683	*A*. *veronii*	2	Yangtze River	*mcr*-3	JANKME000000000

## Results

### *Aeromonas* spp. identification and the characteristics of antibiotic resistance

**Figure 1:**
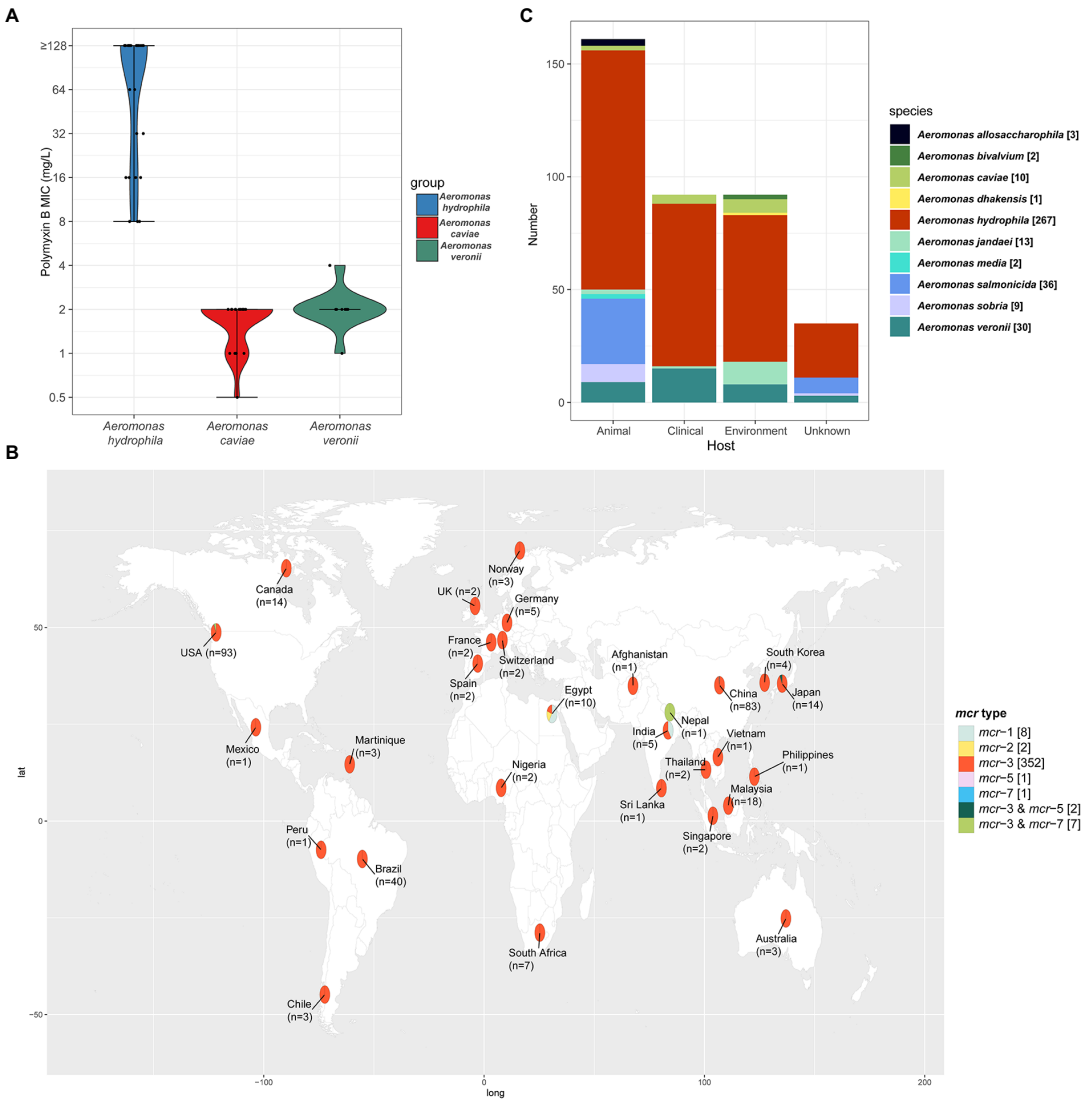
**(A)** The Violin-plot. The MIC of PMB in the three species of the genus *Aeromonas*. The black dot represents the dispersion of MIC value. And the shape of the violin represents the distribution of MICs in the corresponding group. Blue, red, and green indicated the species of *A*. *hydrophila*, *A*. *caviae*, and *A*. *veronii*. **(B)** Geographical distribution of the 326 isolates with definitive location, among all 373 *mcr*-positive *Aeromonas* isolates from the NCBI database. Different *mcr* types of the isolates were labeled with different colors and the number of each *mcr* type is given in square brackets. The number of all the *mcr*-positive isolates in each country was labeled on the world map under the country name. In addition, the location of the 47 *mcr*-positive isolates was still unknown. The world map was created using the corresponding map data with R package ggplot2 v3.3.5 (https://github.com/tidyverse/ggplot2). **(C)** The species and number of *mcr*-positive *Aeromonas* isolates from different sources. In total, 338 *mcr*-positive *Aeromonas* isolates were isolated and identified from the specific host and the sample sources of 35 isolates were still unknown. Different colors indicated the different species of the genus *Aeromonas*.

In order to investigate the distribution of polymyxin resistance in the genus *Aeromonas* bacteria isolated from the aquatic environment, we collected a comprehensive set of samples from the section of the Yangtze River, in Hubei province. In total, 163 gram-negative bacteria strains were isolated and identified by MALDI-TOF MS. Among them, 37.42% (61/163) of the isolates were identified as belonging to *Aeromonas* spp., namely *Aeromonas hydrophila* (*n* = 29), *Aeromonas caviae* (*n* = 21), and *Aeromonas veronii* (*n =* 11). Antimicrobial susceptibility testing was performed to investigate the drug resistance characteristics of 61 *Aeromonas* spp. isolates against fifteen common clinical drugs. 100% of *A*. *hydrophila* isolates exhibited resistance to PMB while 100 and 90.91% (10/11) of *A*. *caviae* and *A*. *veronii* isolates exhibited susceptibility to PMB. 96.72% (59/61), 93.44% (57/61), 90.16% (55/61), 93.44% (57/61), 55.74% (34/61), 65.57% (40/61), 73.77% (45/61), 96.72% (59/61), 50.82% (31/61), 77.05% (47/61), 75.41% (46/61), 83.61% (51/61) of *Aeromonas* spp. strains showed susceptibility to clinically important antibiotic drugs meropenem, imipenem, tigecycline, tazobactam/piperacillin, ciprofloxacin, levofloxacin, aztreonam, amikacin, gentamycin, cefepime, ceftazidime, cefoperazone/sulbactam, respectively. But for the sulfamethoxazole/trimethoprim and tetracycline, the majority of *Aeromonas* spp. remained resistant, with a resistance rate of 67.21% (41/61) and 60.66% (37/61) respectively (Supplementary Table S3). The results of the comparator antimicrobial drugs tested against 61 *Aeromonas* spp. isolates were listed in Table 2. And the MIC_50_ and MIC_90_ for PMB were 128 mg/L and above 128 mg/L in the *A*. *hydrophila* group. However, both the MIC_50_ and MIC_90_ were 2 mg/L in the *A*. *caviae* and *A*. *veronii* groups (Figure 1A). This result shows that the characteristics of PMB resistance in the genus *Aeromonas* vary in different species (*p* < 0.001).

**Table 2:** Comparator antimicrobial agents tested against 61 isolates of *Aeromonas* spp. collected in a section of Yangtze River.

			MIC (mg/L)		
Species (no. of isolates)	Antimicrobial agent				Percentage susceptible (%)
		MIC50	MIC90	MIC range	
*Aeromonas* spp., all (61)	MEN	0.06	0.25	0.015 to 4	96.72
	IPM	0.25	1	0.125 to 4	93.44
	PMB	2	>128	0.5 to >128	50.82
	TGC	0.5	2	<0.125 to 4	90.16
	TZP	2	16	≤1 to 128	93.44
	LVX	1	32	≤0.008 to >64	65.57
	ATM	0.125	16	≤0.06 to >64	73.77
	AMK	4	16	≤0.5 to >128	96.72
	FEP	0.06	32	≤0.015 to 64	77.05
	CIP	1	>64	≤0.004 to >64	55.74
	SXT	>8/152	>8/152	≤0.5/9.5 to >8/152	32.79
	CAZ	0.5	>64	≤0.06 to >64	75.41
	CSL	2	64	0.06 to >128	83.61
	GEN	4	>128	1 to >128	50.82
	TCY	16	128	0.5 to >128	39.34

MEM, meropenem; IPM, imipenem; PMB, polymyxin B; TGC, tigecycline; TZP, piperacillin/tazobactam; LVX, levofloxacin; ATM, aztreonam; AMK, amikacin; FEP, cefepime; CIP, ciprofloxacin; SXT, sulfamethoxazole/trimethoprim; CAZ, ceftazidime; CSL, cefoperazone/Sulbactam; GEN, gentamycin; TCY, tetracycline.

### Identification of *mcr-*positive *Aeromonas* from the aquatic environment

To investigate the prevalence of *mcr* in the genus *Aeromonas* bacteria isolated from the aquatic environment, colony PCR was conducted to screen *mcr* genes using primers as described in materials and methods. Only two *mcr-3* positive *A*. *veronii* strains (M683 and M694, MIC = 2 mg/L) and one *mcr-3* positive *A*. *caviae* S611 (MIC = 0.5 mg/L) were identified, while the rest of the 58 isolates were all negative of *mcr-1* to *mcr-10*, indicating that the prevalence of *mcr* in *Aeromonas* from the section of the Yangtze River is 4.92% (3/61). However, none of the three *mcr*-positive *Aeromonas* strains showed resistance to PMB (MIC <4 mg/L). Conjugation experiments showed that these three *mcr*-positive isolates were not able to transfer the polymyxin resistance phenotype to receipt strain *E*. *coli* J53. S1-PFGE and DNA hybridization were conducted to separate the plasmid of different sizes and locate the genetic position of *mcr-3*. No plasmid was found in the *A*. *caviae* S611. But a plasmid fragment of approximately 5.47 × 10^4^ bp was found in *A*. *veronii* strains (M683 and M694). Southern blotting result suggested that the *mcr-3* was located in the chromosome (Supplementary Figure S1).

**Figure 2:**
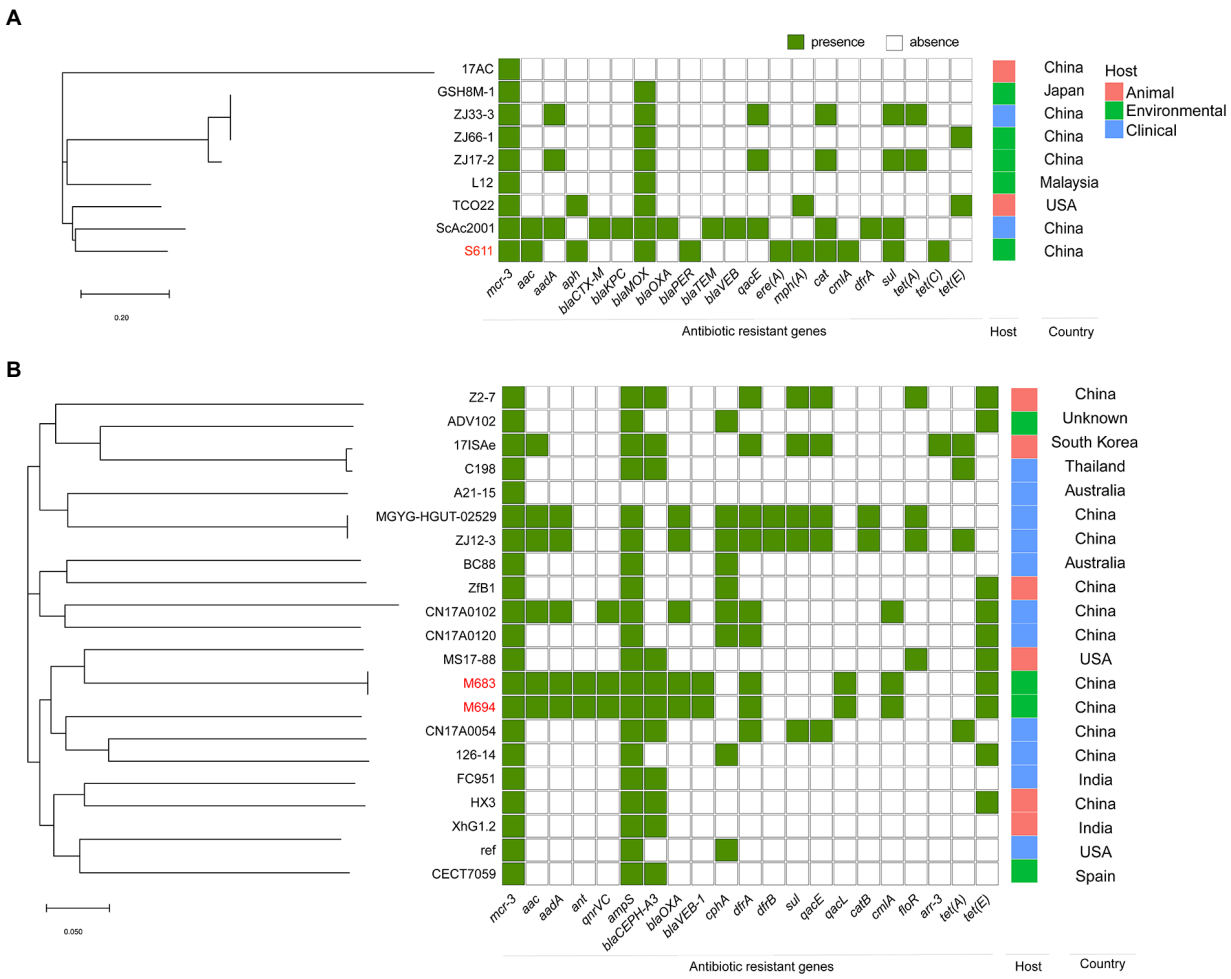
Phylogenetic tree generated from the core-genome sequences of the *mcr*-3-positive isolates identified in this study and other *mcr*-3-positive isolates within the same species randomly selected from GenBank database. Significant antimicrobial resistance genes, country, and host were shown. **(A)** Phylogenetic tree of *mcr*-3-positive *A*. *caviae*. **(B)** Phylogenetic tree of *mcr*-3-positive *A*. *veronii*.

To investigate the characteristics of the epidemiology of *mcr*-positive *Aeromonas* strains, we retrieved all the details of isolates deposited in GenBank as described in materials and methods. The *mcr*-positive *Aeromonas* strains in the GenBank were reported to carry *mcr-1*, *mcr-2*, *mcr-3*, *mcr-5* as well as *mcr-7*, and *mcr-3* was the predominant type in these *mcr*-positive *Aeromonas* strains (47.70%, 352/738). Most of the *mcr*-positive *Aeromonas* were isolated from the United States (24.93%, 93/373) and China (22.25%, 83/373), and *mcr-3* was the main type (94.37%, 352/373) (Figure 1B). Nearly half of the *mcr*-positive *Aeromonas* were isolated from animals (42.63%, 159/373), while 24.40% (91/373) and 23.59% (88/373) of the positive samples were from the environment and clinic (Figure 1C). By the phylogenetic tree analysis, we found that the evolution of *A*. *caviae* S611 isolated from Yangtze River was closely related to *A*. *caviae* SCAc2001 (WUTZ00000000.1) isolated from clinical samples in China. The evolution distance of *A*. *veronii* M683 and M694 isolated from the aquatic environment was quite approximated and closely related to *A*. *veronii* MS-17-88 (GCA_003611985.1) isolated from animal samples in the United States (Figure 2).

### Genome analysis of the *mcr*-positive *Aeromonas* isolates

To fully understand the genetic context of *mcr*-positive *Aeromonas* strains isolated from the section of Yangtze River, we perform WGS with three *mcr-3* positive isolates in this study (*A*. *veronii* M683, M694, and *A*. *caviae* S611) and the contigs were properly assembled.

In the *A*. *veronii* strains M683 and M694, ResFinder was used to reveal the antibiotic resistance genes, such as the aminoglycoside and fluoroquinolone resistance genes (*aac*, *aadA*, *ant*, and *qnrVC*), beta-lactam (*ampS*, *bla*CEPH-A3, *bla*OXA, and *bla*VEB-1), folate pathway antagonist (*dfrA*), quaternary ammonium compound (*qacL*), amphenicol (*cmlA*), and tetracycline antibiotic genes (*tet*(E)). In the *A*. *caviae* S611, the following antibiotic resistance genes were predicted, *aac*, *aph*, *bla*MOX, *bla*PER, *sul*, macrolide resistance genes (*mph*(A) and *ere*(A)), *cat*, *cmlA*, and *tet*(C) (Figure 2).

**Figure 3:**
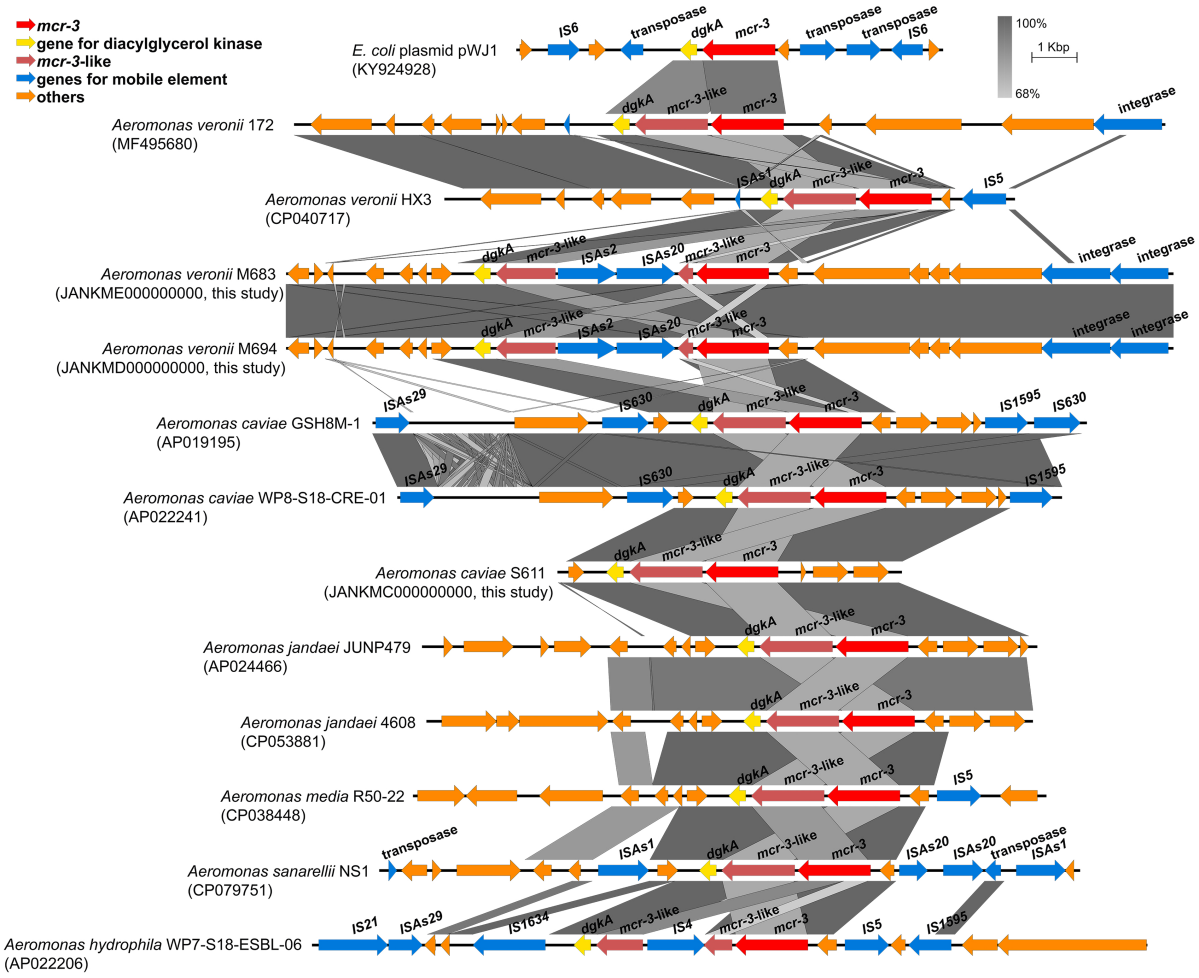
Schematic representation and comparison of the genetic environment of the *mcr*-3-flanking region in each genomic backbone type. Arrows indicate the direction of transcription of each gene, and different genes are shown in different colors. Regions of above 68% nucleotide sequence identify are shaded grey.

The nucleotide sequence of *mcr-3* in the *A*. *caviae* S611 showed 100% identity with *mcr-3*.*16* (NG_060517.1). A 1626-bp *mcr-3*-like gene lay downstream of the *mcr-3*. Its nucleotide sequence showed 83.03 and 67.32% identity to *mcr-3*.*16* and lipid A phosphoethanolamine transferase gene (*eptA*) in the *A*. *caviae* S611. Downstream region of the *mcr-3*-like gene lay *dgkA* encoding diacylglycerol kinase, a gene arrangement (*mcr-3*-*mcr-3*-like) that is often observed in many *mcr-3* positive *Aeromonas* strains. But *A*. *veronii* strains M683 and M694 were the exception. Their *mcr-3*-*mcr-3*-like segment was separated by the insertion sequence of IS*As20* (1,674 bp) and IS*As2* (1,084 bp) (Figure 3). This is the first report of the truncated *mcr-3*-*mcr-3*-like segment in the *mcr*-3 positive *A*. *veronii* strains. Both of the two 1,623-bp *mcr-3* variant genes of M683 and M694 exhibited 99.94% nucleotide sequence identity to the *mcr-3*.*32* (NG_070770.1), and the corresponding proteins exhibited 99.81% amino acid sequence identity to MCR-3.32 (WP_188331891.1). The two novel *mcr-3* variant genes of *A*. *veronii* M683 and M694 were termed as *mcr-3*.*42*. And the accession numbers were OP297669 and OP297670, respectively. The discovery of *mcr-3*.*42* genes in *A*. *veronii* was the first report about the *mcr-3* positive *A*. *veronii* strains isolated from the aquatic environment in China.

### The sequence variants of genes related to polymyxin resistance in different *Aeromonas* species

Although *mcr-3* gene were detected in some *Aeromonas* strains, these bacteria did not show high colistin resistance. And other *Aeromonas* strains with high colistin resistance did not possess *mcr* gene. Therefore, we suspected that the *mcr* gene might not be the primary cause for the colistin resistance diversity among *Aeromonas*. In order to investigate the possible reason for that, we screened 12 *mcr*-negative *Aeromonas* strains in three species with different levels of PMB sensibility (MIC of 4 *A*. *hydrophila* isolates was ≥128 mg/L, MIC of 4 *A*. *caviae* and 4 *A*. *veronii* isolates were ≤ 2 mg/L). Whole genome sequencing was performed to analyze the differences in the gene sequences of TCS such as PhoP/PhoQ, EnvZ/OmpR TCS, and MlaF in these 12 *Aeromonas* strains. And amino acids analysis and protein structure prediction was performed as described in materials and methods.

**Figure 4:**
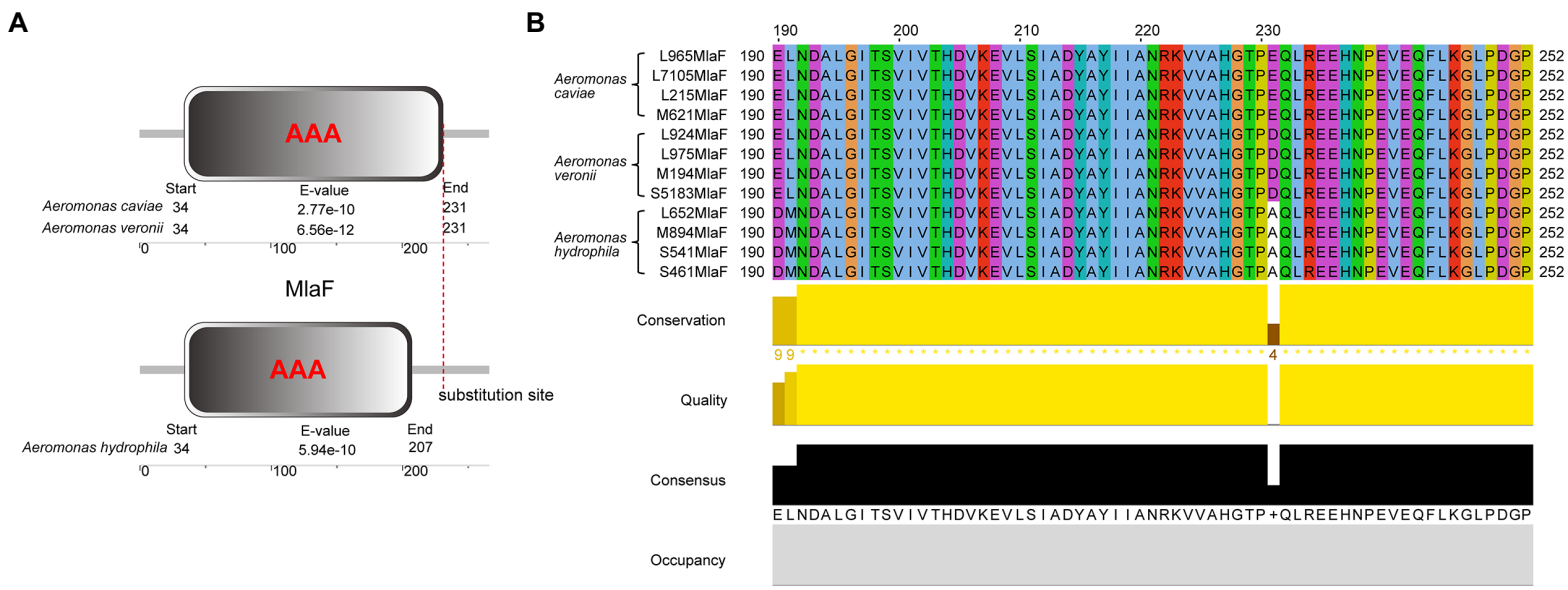
The structure and multiple sequence alignment of MlaF protein. **(A)** The structure of MlaF domain. **(B)** The multiple sequence alignment of MlaF from 225 position to the end in three species of the genus *Aeromonas*. The beautification of the image was used the Jalview 2.11.1.7 and the amino acid color was used Clustalx.

**Figure 5:**
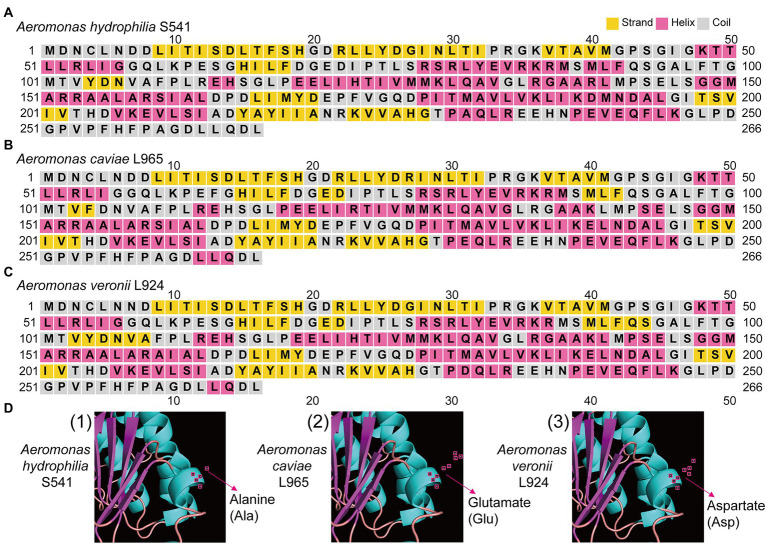
The structure of MlaF protein in three *Aeromonas* species. **(A)** The predicted secondary structure of MlaF protein in *A*. *hydrophilia* S541. **(B)** The predicted secondary structure of MlaF protein in *A*. *caviae* L965. **(C)** The predicted secondary structure of MlaF protein in *A*. *veronii* L924. **(D)** The predicted three-dimensional structure of MlaF protein in corresponding strains. The amino acid of 231 position of MlaF was pointed by red arrow.

We compared the genome sequences of *A*. *hydrophila* with *A*. *caviae* and *A*. *veronii*, colistin resistance gene *arnBCADTEF* was only found in *A*. *hydrophila*, implying that *arnBCADTEF* operon might be the possible cause for the variety of colistin sensitivity among these species. We also screened the nucleic acids differences in the TCS PhoP/PhoQ, EnvZ/OmpR, and MlaF. Many base mutations were found in these genes in *A*. *hydrophila*, but most of the mutations did not change the amino acid sequences. For example, compared with *A*. *caviae* genome sequence, *A*. *hydrophila* possessed 12 site mutations in *phoP*, 53 site mutations in *phoQ*, 7 site mutations in *ompR*, 32 site mutations in *envZ*, and 32 site mutations in *mlaF*. While compared with *A*. *veronii*, there were 12, 93, 10, 55, and 5 site mutations in these genes (Supplementary Table S4). In order to evaluate whether these mutations would impact the protein function, PROVEAN software was used to predict the result of these mutations (Table 3). For *A*. *hydrophila*, PhoP amino acid substitution in Q39G, R69S/T, V153T/A, PhoQ in P82R, Y233F, OmpR in F30V, EnvZ in G108K, Q198L, Q302Y, L339Q, P369A, H407Y, and MlaF in S63F, A231E/D were supposed to cause disfunction. But secondary structure alignment showed that the AAA domain in MlaF of *A*. *hydrophila* (174aa) was shorter than that of *A*. *veronii* and *A*. *caviae* (198aa) (Figure 4). And protein structures predictions of MlaF based on the model proteins showed that MlaF of *A*. *hydrophila* lacked the C-terminal helix structure that exists in both MlaF of *A*. *veronii* and *A*. *caviae* (Figure 5).

**Table 3:** Amino acid variations in functional domains of PhoP/PhoQ, EnvZ/OmpR, and MlaF in *Aeromonas*.

Species	MIC	Strain	PhoP			PhoQ	OmpR	EnvZ						MlaF		
			REC	Trans_reg_c		Unknown	REC	Unknown	HAMP	Unknown		HATPase_c		AAA	AAA/unknown	
			39	69	153	82	233	30	108	198	302	339	369	407	63	231
*A*. *hydrophila*	>128	L652	Q	R	V	P	Y	F	G	Q	Q	L	P	H	S	A
*A*. *hydrophila*	>128	M894	Q	R	V	P	Y	V	G	Q	Q	L	P	H	S	A
*A*. *hydrophila*	>128	S461	Q	R	V	P	Y	F	G	Q	Q	L	P	H	S	A
*A*. *hydrophila*	>128	S541	Q	R	V	P	Y	F	G	Q	Q	L	P	H	S	A
*A*. *caviae*	1	L215	Q	S	T	P	Y	F	K	Q	Q	L	A	Y	S	E
*A*. *caviae*	1	L965	Q	T	T	P	Y	F	K	Q	Q	L	A	H	F	E
*A*. *caviae*	2	L7105	Q	T	T	P	Y	F	K	Q	Q	L	A	H	F	E
*A*. *caviae*	1	M621	Q	T	T	P	Y	F	K	Q	Q	L	A	H	S	E
*A*. *veronii*	2	L924	G	R	A	R	F	F	G	L	Y	L	P	H	S	D
*A*. *veronii*	1	L975	G	R	A	R	F	F	G	L	Y	L	P	H	S	D
*A*. *veronii*	2	M194	G	R	A	R	F	F	G	L	Y	Q	P	H	S	D
*A*. *veronii*	2	S5183	G	R	A	R	F	F	G	L	Y	Q	P	H	S	D

Q, glutamine (Gln); G, glycine (GLy); R, arginine (Arg); S, serine (Ser); T, threonine (Thr); V, valine (Val); A, alanine (Ala); P, proline (Pro); Y, tyrosine (Tyr); F, phenylalanine (Phe); K, lysine (Lys); L, leucine (Leu); H, histidine (His); E, glutamic acid (Glu); D, aspartic acid (Asp).

**Figure 6:**
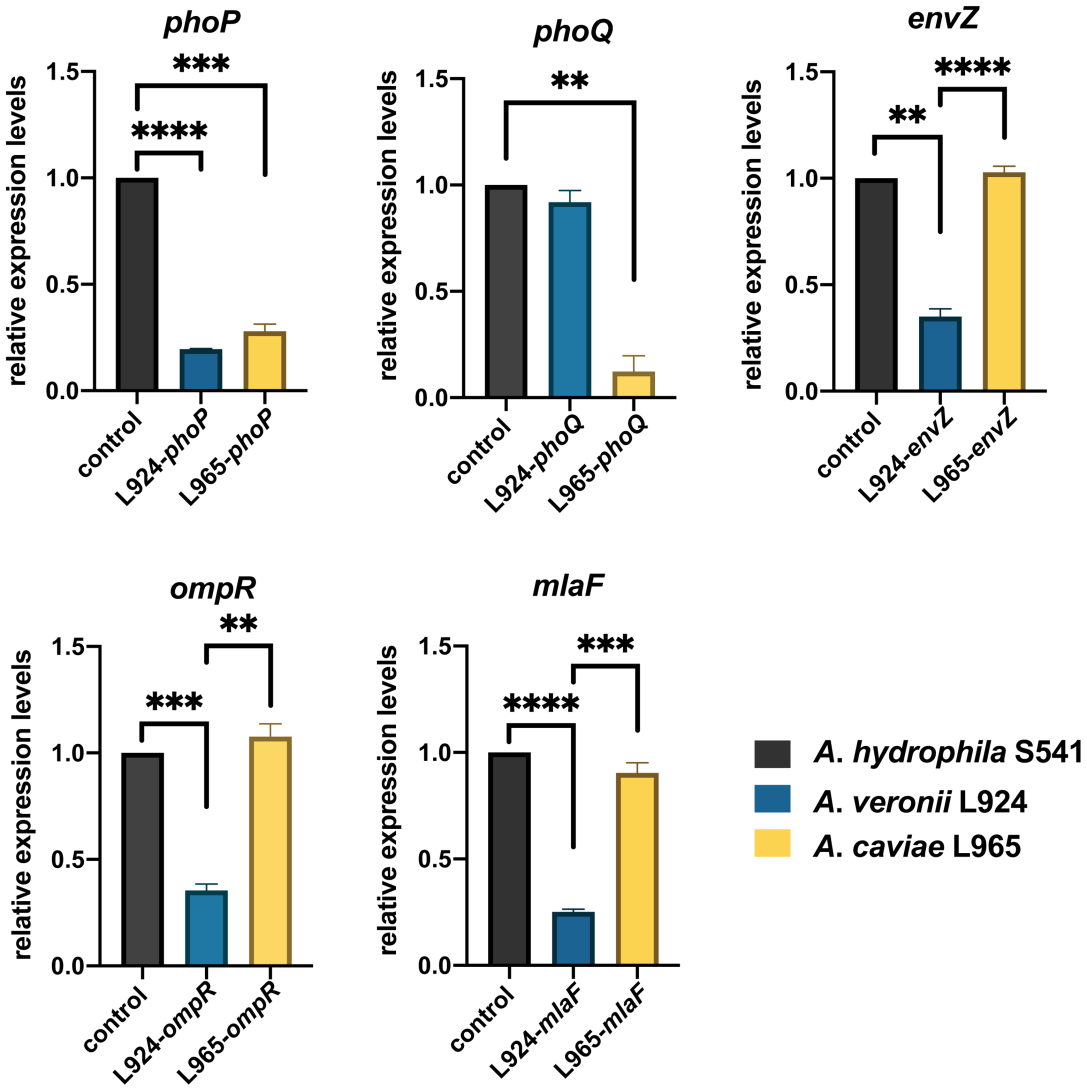
Gene expression of *A*. *hydrophila* S541, *A*. *veronii* L924, *A*. *caviae* L965. Relative expression levels of genes were determined using the 2^−ΔΔct^ method. Error bars represent the standard deviation of three biological replicates. **p < 0.01; ***p < 0.001, ****p < 0.0001.

In order to investigate whether expression levels of these TCS were involved with discrepancy of polymyxin resistance, we selected one strain as a representative strain from each of the three species with the fewest intraspecific mutations based on multiple alignments of TCS genes to perform qRT-PCR. Transcriptional levels of *phoP/Q*, *envZ/ompR*, and *mlaF* in *A*. *hydrophila* S541 (PMB MIC>128 mg/L), *A*. *caviae* L965 (PMB MIC = 1 mg/L), and *A*. *veronii* L924 (PMB MIC = 2 mg/L) were detected by qRT-PCR. *A*. *hydrophila* S541 was acted as the control. As shown in Figure 6, compared with *A*. *hydrophila* S541, transcriptional levels of *phoP*, *envZ*, *ompR*, and *mlaF* decreased in *A*. *veronii* L924, while only expression levels of *phoP*/*Q* decreased in *A*. *caviae* L965. We inferred that upregulated TCS PhoPQ in the colistin-resistant strain *A*. *hydrophila* S541 might participate in the diversity of colistin resistance among the genus *Aeromonas*.

## Discussion

Water, especially the sewage regeneration system is the medium for the spread of *Aeromonas* spp. (McLellan et al., 2010; Pablos et al., 2011). The epidemiological study of *Aeromonas* spp. and sewage metagenomic studies have reported that *A*. *caviae* strains could be isolated from drinking water samples and diarrhea feces. Chen *et al* confirmed that horizontal gene transfer (HGT) was the dominant way to spread drug resistance in the water phase (Chen et al., 2019). The aquatic environment could be a reservoir for the transmission of polymyxin-resistant bacteria. For example, the aquatic bacterium *Shewanella* and its associated aquaculture are the reservoir for the dissemination of *mcr-4* (Zhang et al., 2019a,b). It has been reported that *Aeromonas* may be the container for *mcr-3* and *mcr-7*, although it is not the inherent host of *mcr-3* (Parker and Shaw, 2011; Eichhorn et al., 2018; Shen et al., 2018). According to available research reports, it reveals that variants of *mcr-1* to *mcr-10* exhibit selectivity to some specific species. For example, *mcr-3* and *mcr-7* genes have been reported to locate on the chromosomes in the genus *Aeromonas* such as *A*. *hydrophila*, *A*. *veronii*, *A*. *caviae*, *A*. *jandaei* (Parker and Shaw, 2011; Ling et al., 2017). Our study proved this and showed that the genus *Aeromonas* and aquatic environment might be the potential container and reservoir of *mcr-3*.

Compared with abundant studies focused on polymyxin-resistant bacteria from clinics, less work has been carried out in the aquatic environment in China (Yang et al., 2017; Tuo et al., 2018; Pan et al., 2022). By phylogenetic tree analysis, we found that three *mcr-3* positive *Aeromonas* strains isolated from Yangtze River have a close evolution distance with clinical samples from China and animal samples from the United States, showing a wide spread of the *mcr-3* in *Aeromonas* strains. In this study, susceptibility of PMB between different species in the genus *Aeromonas* exhibited a distinct discrepancy according to the results of MIC_50_ and MIC_90_ (*p* < 0.001). No *mcr* gene was found in *A*. *hydrophila*, but all the strains showed high-level resistance to PMB (8 mg/L ≤ MIC ≤128 mg/L). Three *mcr-3* positive strains were found in the *A*. *caviae* and *A*. *veronii* strains while most *A*. *caviae* and *A*. *veronii* isolates were susceptible to PMB (0.5 mg/L ≤ MIC ≤4 mg/L). Gene *mcr-3* in this study were all located on chromosomes as other studies have reported (Ling et al., 2017; Liu et al., 2020; Wang et al., 2021). This is the first report about the identification of *mcr-3*.*42* positive *A*. *veronii* strains isolated from the aquatic environment in China and was also the first report of the variant of polymyxin resistance phenotype in different species of the genus *Aeromonas*.

Genetic analysis in the surrounding gene–environment of the *mcr-3* positive isolates found that the *mcr-3*-like was truncated by IS*As20* (1,674 bp) and IS*As2* (1,084 bp) in two *A*. *veronii* isolates, leading to the separation of *mcr-3*-*mcr-3*-like segment. The truncated *mcr-3*-*mcr-3*-like segment is seldomly observed in the genus *Aeromonas*. Likewise, the *mcr-3*-like gene was also divided into two fragments by IS*4* in the *A*. *hydrophila* WP7-S18-ESBL-06. It indicated that the *mcr-3*-*mcr-3*-like segment was not so conserved that it could be divided by IS. In the upstream region of *mcr-3*, there also lied an IS. Wang *et al* have reported a novel transposon Tn6518 in the *A*. *veronii* w55, composing the genetic element IS*As2*-IS*Ahy2*-IS*As20*-*mcr-3*.*6*-*mcr-3*-like-*dgkA*-IS*As2* (Wang et al., 2020). In *A*. *veronii* FC951, *mcr-3*.*19* was inserted by an IS*As18* and become inactive, making the *A*. *veronii* FC951 susceptible to colistin (Ragupathi et al., 2020). Some IS family members, including IS*3*, IS*30*, IS*110*, IS*26*, and IS*CR1* elements, utilize circular DNA intermediates containing accessory genes to undergo gene translocation *via* copy-and-paste mechanisms. We supposed that the IS-gene-*mcr-3*-*mcr-3*-like-truncated-IS would be conducive to the spread of colistin resistance in different strains and leads to the dissemination of *mcr-3* in the aquatic environment. The three *mcr*-positive strains isolated from the Yangtze River were all susceptible to PMB. Among the reported *mcr-3* positive *Aeromonas*, most of the *A*. *veronii* and *A*. *caviae* were sensitive to polymyxins, while *A*. *hydrophila* were highly resistant to polymyxin (Ling et al., 2017; Shen et al., 2018; Hatrongjit et al., 2020; Huang et al., 2020; Wang et al., 2021). Therefore, we speculated that *mcr-3* might not be the main factor leading to the resistance to polymyxin among *Aeromonas*. One possible reason could be that the expression of *mcr-3* might not be activated due to the loss of expression elements during the process of the insertion of IS*As20* and IS*As2*.

Genome analysis of the isolates discovered that *arnBCADTEF* operon only existed in *A*. *hydrophila* genomes but not in *A*. *caviae* and *A*. *veronii* genomes, which might be a reason for the different phenotypes of polymyxin susceptibility among these *Aeromonas* strains. It has been reported that *A*. *hydrophila* could thrive in a complex colistin environment with the help of EnvZ/OmpR TCS and MlaF (Liu et al., 2021). We used these as the entry point and tried to investigate the possible cause for diversity in the colistin resistance in the *Aeromonas* genus by comparing gene sequences of PhoP/PhoQ TCS, EnvZ/OmpR TCS, and MlaF. Though some site mutations were found in PhoP/PhoQ and EnvZ/OmpR TCS, this nucleic acid substitution did not change the conformation structure of these proteins. Only nucleic acid changes in MlaF were predicted to impact the length of its domain. MlaF was one of the proteins of the Mla system that has played an essential role in phospholipid (PL) transport and constituted the inner membrane ABC transporter complex, MlaFEDB (Coudray et al., 2020). It is reported that MlaF was involved in the antibiotic resistance in *A*. *hydrophila* and the mutation in *mlaF* (*mlaF_D173A_*) confers high-level colistin resistance *via* upregulation of the Mla system (Liu et al., 2021; Zhou et al., 2021). In this study, the substitution at the 231 position of the MlaF was predicated to be deleterious and this substitution may affect the biological function when the acidic amino acid that is E or D in the susceptible strains (*A*. *caviae* and *A*. *veronii*) is substituted for the neutral amino acid that is A in the resistant strains (*A*. *hydrophila*). We infer that the substitution at 231 position in MlaF in the susceptible strains (*A*. *caviae* and *A*. *veronii*) may alter the steric hindrance and may be related to the polymyxin resistance in different species. In addition, MlaF subunit possesses a unique ~25 aa C-terminal extension (CTE) forming a domain-swapped reciprocal ‘handshake’ that interacts with the adjacent MlaB to fulfill the function (Kolich et al., 2020). And results of the secondary structure alignment of MlaF showed that helix existed in the susceptible strains (*A*. *caviae* and *A*. *veronii*, Figures 5B,C), not in the resistant strains (*A*. *hydrophila*, Figure 5A), which might have an influence on the interaction with MlaB, hence affecting the Mla system. In addition, the expression level of *mlaF* in *A*. *hydrophila* S541 and *A*. *caviae* L965 was much higher than that in *A*. *veronii* L924 (Figure 6). We supposed that the higher expression of *mlaF* might cause an increased level of PL and maintain the membrane homeostasis so as to withstand the polymyxin. Thus, we inferred that the amino acids differences in MlaF protein and its expression levels among different *Aeromonas* species could affect the function of transportation in the Mla system which is involved in the synthesis of membrane components and contribute to the resistance to polymyxins. In general, we speculated that the existence of *arnBCADTEF* and sequence differences in MlaF might contribute to the variety of polymyxin susceptibility in different species of the genus *Aeromonas*.

In sum, this is the first report describing the variety in the phenotype of polymyxin susceptibility in different species of the genus *Aeromonas*. And our study also identified two novel *mcr-3*.*42* positive *A*. *veronii* strains and one *mcr-3*.*16* positive *A*. *caviae* strain in the aquatic environment in China for the first time. We provided pieces of evidence of the possible mechanism for the different polymyxin susceptibility in different species of the genus *Aeromonas*, but the exact mechanism deserved further research.

## Data availability statement

The datasets presented in this study can be found in online repositories. The names of the repository/repositories and accession number(s) can be found in the article/Supplementary material.

## Author contributions

QL conceived and designed the experiments. LX, HF, and JF performed the experiments. YY, QL, and HF contributed to the reagents, materials, and analysis tools. LX wrote the original draft. QL and FW checked and revised the manuscript. All authors contributed to the article and approved the submitted version.

## Funding

This research was funded by the National Key R&D Program of China (2021YFC2300302), National Natural Science Foundation of China (31900021), National Science Foundation of Zhejiang Province, China (LY20H190002), and Research Project of Jinan Microecological Biomedicine Shandong Laboratory (JNL-2022031C).

## Conflict of interest

The authors declare that the research was conducted in the absence of any commercial or financial relationships that could be construed as a potential conflict of interest.

## Publisher’s note

All claims expressed in this article are solely those of the authors and do not necessarily represent those of their affiliated organizations, or those of the publisher, the editors and the reviewers. Any product that may be evaluated in this article, or claim that may be made by its manufacturer, is not guaranteed or endorsed by the publisher.

## Footnotes

^1^ https://eucast.org/clinical_breakpoints/

^2^ https://ncbi.nlm.nih.gov/orffinder/

^3^ http://rast.nmpdr.org/

^4^ http://github.com/tseemann/prokka

^5^ http://genomicepidemiology.org/

^6^ https://www.ebi.ac.uk/Tools/msa/clustalo/

^7^ https://www.ncbi.nlm.nih.gov/data-hub/genome/

^8^ https://www.ncbi.nlm.nih.gov/pathogens/isolates/

^9^ https://www.ebi.ac.uk/Tools/msa/clustalo/

^10^ http://provean.jcvi.org/index.php

^11^ http://smart.embl.de/

^12^ http://bioinf.cs.ucl.ac.uk/psipred/

^13^ https://swissmodel.expasy.org/
