# Supplementary material for: The variants of polymyxin susceptibility in different species of genus Aeromonas
Source: Front Microbiol. 2022 Oct 25;13:1030564. doi: 10.3389/fmicb.2022.1030564 (PMC9642839; doi:10.3389/fmicb.2022.1030564)
Supplement: Supplementary file 2 [file Data_Sheet_1.docx]

Supplementary Material

# Supplementary Figures and Tables

## Supplementary Figures


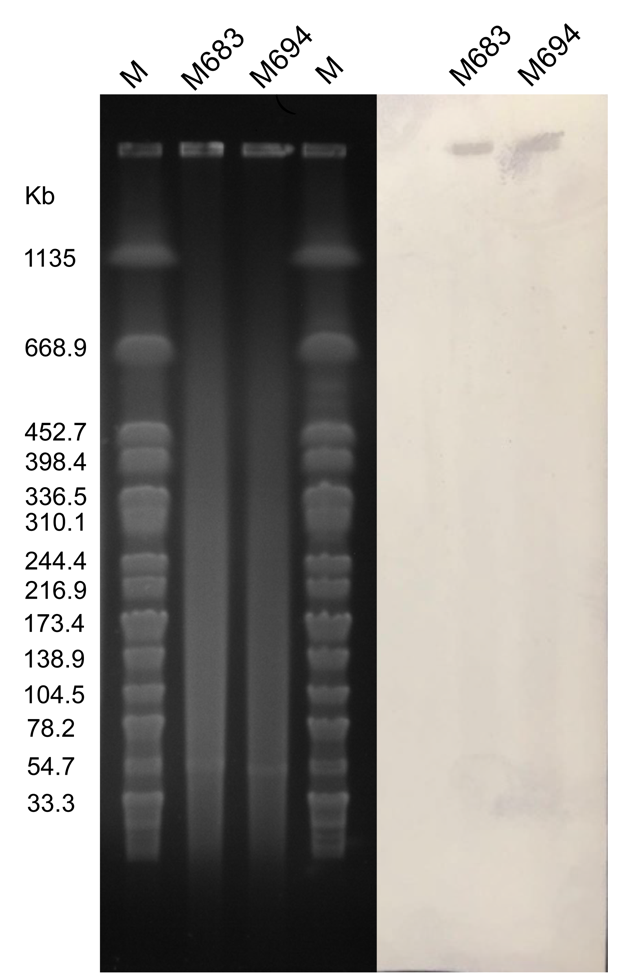


**Supplementary Figure 1.** S1-PFGE profiles (left) and Southern blot (right) analysis with an *mcr-3* specific probe of *mcr-3* harboring isolates, with *Salmonella enterica* serovar Branderup as a molecular mass marker. The names of the isolates are shown in the first line.

## Supplementary Tables

**Table S1. The primer sequence of *mcr*-1 to *mcr*-10 and 16SrDNA sequencing.**

| gene | primer | Sequence (5’-3’) | product (bp) | reference |
| --- | --- | --- | --- | --- |
| *mcr*-1 | *mcr*-1_F | TCCAAAATGCCCTACAGACC | 205 | This study |
|  | *mcr*-1_R | GCCACCACAGGCAGTAAAAT |  |  |
| *mcr*-2 | *mcr*-2_F | CCTTTTGTGCTGATGGGTTT | 279 | This study |
|  | *mcr*-2_R | ATTTTGGAGCATGGTGGTGT |  |  |
| *mcr*-3 | *mcr*-3_F | CTTGCTGAACCAATCCCATT | 347 | This study |
|  | *mcr*-3_R | CCATCGTTCTCCTTCCACAAAA |  |  |
| *mcr*-4 | *mcr*-4_F | GATCCGAAGCTGTGTTCTG | 426 | This study |
|  | *mcr*-4_R | GCCAGCATTGGTACGCTAGT |  |  |
| *mcr*-5 | *mcr*-5_F | GGTTGGCCGAGAAGATAACA | 522 | This study |
|  | *mcr*-5_R | ATGTTGCCAGAAGGTCCAAC |  |  |
| *mcr*-6 | *mcr*-6_F | AGCTATGTCAATCCCGTGAT | 252 | (Borowiak et al., 2020) |
|  | *mcr*-6_R | ATTGGCTAGGTTGTCAATC |  |  |
| *mcr*-7 | *mcr*-7_F | GTCAGTTACGCCATGCTCAA | 791 | This study |
|  | *mcr*-7_R | TTCTTGTCGCAGAACTGTGG |  |  |
| *mcr*-8 | *mcr*-8_F | AAACTGAACCCGGTACAACG | 943 | This study |
|  | *mcr*-8_R | GCCATAGCACCTCAACACCT |  |  |
| *mcr*-9 | *mcr*-9_F | GCGGTTGTAAAGGCGTATGT | 635 | This study |
|  | *mcr*-9_R | CAAATCGCGGTCAGGATTAT |  |  |
| *mcr*-10 | *mcr*-10_F | GGACCGACCTATTACCAGCG | 366 | (Lei et al., 2020) |
|  | *mcr*-10_R | GGCATTATGCTGCAGACACG |  |  |
| 16S rRNA | 515F | GTGYCAGCMGCCGCGGTAA | ~390 | (Parada et al., 2016) |
|  | 806R | GGACTACNVGGGTWTCTAAT |  | (Walters et al., 2016) |

**Table S2. The primer sequence of qPCR.**

| gene | primer | sequence (5’-3’) | product (bp) |
| --- | --- | --- | --- |
| 16SrRNA | 16SrRNA _F | GTGCCTTCGGGAATCAGAAC | 111 |
|  | 16SrRNA _R | GTGCTGGCAACAAAGGACAG |  |
| *phoP* | phoP_F | GGCGACTACGAGCTGGATCT | 108 |
|  | phoP_R | GGCGTTGCGCATCAGGT |  |
| *phoQ* | phoQ_F | TGGATACCCTGATCTGCC | 112 |
|  | phoQ_R | CCCAGATACTTCATCAGATC |  |
| *envZ* | envZ_F | AGCGCAACCTGATGATGG | 115 |
|  | envZ_R | ATTGATGCTCTCCTTGAGGT |  |
| *ompR* | ompR_F | GTGCTCAAGGTGCTGGT | 115 |
|  | ompR_R | AGACCTGCACGTCGATGC |  |
| *mlaF* | mlaF_F | CCGACCTGACCTTCAGTCAC | 109 |
|  | mlaF_R | GTCTTGCCGATGCCGCT |  |

**Table S3. MIC profiles of 61 *Aeromonas* spp. strains in common clinical antibiotics.**

| isolates | species | the MICs of antibiotics | | | | | | | | | | | | | | |
| --- | --- | --- | --- | --- | --- | --- | --- | --- | --- | --- | --- | --- | --- | --- | --- | --- |
|  |  | MEM | IPM | PMB | TGC | TZP | LVX | ATM | AMK | FEP | CIP | SXT | CAZ | CSL | GEN | TCY |
| L112 | *Aeromonas hydrophila* | 0.015 | 0.5 | 64 | ＜0.125 | 2 | 4 | 0.125 | 4 | 0.125 | 32 | ＞8/152 | 0.5 | 2 | 2 | ＞128 |
| L1132 | *Aeromonas hydrophila* | 0.03 | 1 | 32 | ＜0.125 | 2 | 4 | 0.125 | 4 | 0.03 | 32 | ＞8/152 | 0.5 | 2 | 2 | ＞128 |
| L241 | *Aeromonas hydrophila* | 0.03 | 0.25 | 8 | 1 | 8 | 32 | 32 | 16 | 16 | 32 | 1/19 | ＞64 | 32 | 2 | 32 |
| L321 | *Aeromonas hydrophila* | 0.125 | 4 | ＞128 | 1 | ≤1 | 2 | 0.125 | 4 | 0.06 | 8 | 4/76 | 0.5 | 0.5 | 128 | 4 |
| L341 | *Aeromonas hydrophila* | 0.125 | 4 | ＞128 | 1 | ≤1 | 2 | 0.125 | 4 | 0.06 | 4 | 4/76 | 0.5 | 0.5 | 64 | 4 |
| L3113 | *Aeromonas hydrophila* | 0.03 | 0.5 | ＞128 | 1 | 2 | 0.5 | 0.125 | 2 | 0.03 | 0.5 | ≤0.5/9.5 | 0.25 | 0.5 | 2 | 32 |
| L3164 | *Aeromonas hydrophila* | 0.03 | 0.5 | 128 | 0.125 | 2 | 0.5 | 0.125 | 8 | 0.03 | 0.5 | ＞8/152 | 0.25 | 4 | ＞128 | 32 |
| L3215 | *Aeromonas hydrophila* | 0.03 | 0.5 | 128 | 0.125 | 2 | 0.5 | 0.125 | 2 | 0.03 | 0.5 | ＞8/152 | 0.25 | 4 | 128 | 32 |
| L652 | *Aeromonas hydrophila* | 0.03 | 1 | ＞128 | 0.25 | ≤1 | 0.5 | 0.5 | ＞128 | 0.25 | 0.5 | ＞8/152 | 16 | 2 | ＞128 | 16 |
| L662 | *Aeromonas hydrophila* | 0.06 | 1 | ＞128 | 0.25 | 2 | 0.5 | ≤0.06 | 4 | 0.03 | 0.5 | ＞8/152 | 0.25 | 0.5 | 128 | 1 |
| L672 | *Aeromonas hydrophila* | 0.015 | 0.5 | ＞128 | 0.5 | ≤1 | 4 | ≤0.06 | 4 | 0.03 | 8 | 2/38 | 1 | 4 | ＞128 | 32 |
| L6115 | *Aeromonas hydrophila* | 0.015 | 0.25 | ＞128 | 0.25 | ≤1 | 0.5 | ≤0.06 | 2 | 0.03 | 0.5 | 1/19 | 0.25 | 0.5 | 64 | 0.5 |
| L6135 | *Aeromonas hydrophila* | 0.015 | 0.5 | 128 | 0.5 | ≤1 | 0.5 | 0.125 | 4 | 0.03 | 0.5 | ＞8/152 | 0.125 | 2 | 128 | 16 |
| L821 | *Aeromonas hydrophila* | 0.06 | 0.25 | 32 | 0.25 | ≤1 | 8 | ≤0.06 | 2 | 0.03 | 8 | ＞8/152 | 0.25 | 2 | 2 | 16 |
| L831 | *Aeromonas hydrophila* | 0.03 | 0.25 | 16 | 0.125 | ≤1 | 0.25 | ≤0.06 | 1 | 0.03 | 0.125 | ＞8/152 | 0.125 | 0.25 | 1 | 16 |
| L911 | *Aeromonas hydrophila* | 0.06 | 0.25 | 64 | 0.25 | 2 | 0.25 | 0.125 | 2 | 0.03 | 0.125 | ＞8/152 | 0.25 | 0.25 | 2 | 16 |
| L931 | *Aeromonas hydrophila* | 0.03 | 1 | 8 | 0.25 | 64 | 0.5 | 0.125 | 2 | 0.06 | 0.125 | ＞8/152 | 0.25 | 4 | 2 | 32 |
| L941 | *Aeromonas hydrophila* | 2 | 1 | 16 | 0.25 | 2 | 0.5 | 0.125 | 16 | 0.25 | 0.5 | ＞8/152 | 1 | 4 | 4 | 32 |
| S211 | *Aeromonas hydrophila* | 0.125 | 0.25 | 128 | 4 | 4 | 64 | 0.125 | 2 | 0.5 | 64 | ＞8/152 | 1 | 4 | 2 | 128 |
| S221 | *Aeromonas hydrophila* | 0.125 | 0.125 | 128 | 4 | 4 | ＞64 | 0.125 | 2 | 0.5 | ＞64 | ＞8/152 | 1 | 4 | ＞128 | 128 |
| S231 | *Aeromonas hydrophila* | 0.125 | 0.25 | 128 | 4 | 4 | ＞64 | 0.125 | 2 | 0.5 | ＞64 | ＞8/152 | 1 | 4 | ＞128 | 128 |
| S461 | *Aeromonas hydrophila* | 0.25 | 0.5 | ＞128 | 1 | 2 | ≤0.008 | 0.125 | 2 | 0.03 | 0.015 | 1/19 | 0.125 | 0.25 | 16 | 0.5 |
| S541 | *Aeromonas hydrophila* | 0.125 | 0.5 | ＞128 | 0.5 | 2 | ≤0.008 | 0.125 | 4 | 0.03 | ≤0.004 | ≤0.5/9.5 | 0.125 | 0.06 | 2 | 0.5 |
| S551 | *Aeromonas hydrophila* | 0.25 | 0.5 | ＞128 | 0.5 | ≤1 | ≤0.008 | 0.125 | 4 | ≤0.015 | ≤0.004 | ≤0.5/9.5 | 0.125 | 0.25 | 2 | 0.5 |
| S5234 | *Aeromonas hydrophila* | 0.06 | 0.5 | 128 | 0.25 | 2 | 0.5 | 0.125 | 2 | 0.06 | 0.5 | ≤0.5/9.5 | 0.25 | 0.5 | 1 | 1 |
| S641 | *Aeromonas hydrophila* | 0.03 | 0.25 | 16 | 0.25 | ≤1 | 0.25 | ≤0.06 | 16 | ≤0.015 | 0.125 | ≤0.5/9.5 | 0.125 | 0.25 | 16 | 0.5 |
| S651 | *Aeromonas hydrophila* | 0.03 | 0.25 | 8 | 0.125 | ≤1 | 0.25 | ≤0.06 | ＞128 | ≤0.015 | 0.125 | ≤0.5/9.5 | 0.125 | 0.06 | 128 | 1 |
| M894 | *Aeromonas hydrophila* | 4 | 4 | ＞128 | 0.5 | ≤1 | 0.06 | 0.125 | 4 | 0.06 | 0.008 | ≤0.5/9.5 | 0.25 | 2 | 2 | 2 |
| M941 | *Aeromonas hydrophila* | 0.03 | 0.25 | 16 | 1 | ≤1 | 1 | 16 | 4 | 2 | 1 | ＞8/152 | ＞64 | 1 | 2 | 16 |
| L215 | *Aeromonas caviae* | 0.015 | 0.125 | 1 | 1 | 8 | 32 | 16 | 16 | 32 | 32 | ＜0.5/9.5 | 4 | 32 | 4 | 32 |
| L282 | *Aeromonas caviae* | 0.03 | 0.5 | 2 | 2 | 8 | 64 | 16 | 8 | 64 | ＞64 | ＞8/152 | 8 | 32 | 4 | ＞128 |
| L2102 | *Aeromonas caviae* | 0.06 | 0.5 | 2 | 1 | 64 | 32 | 2 | 4 | 0.5 | 64 | ＞8/152 | 16 | ＞128 | 128 | 128 |
| L6125 | *Aeromonas caviae* | 0.015 | 0.25 | 1 | 0.5 | ≤1 | 2 | 2 | 16 | 2 | 1 | ＞8/152 | 32 | 8 | 128 | 16 |
| L6145 | *Aeromonas caviae* | 0.125 | 1 | 2 | 0.5 | 128 | 4 | 0.125 | 8 | 16 | 2 | ＞8/152 | ＞64 | ＞128 | ＞128 | 32 |
| L7105 | *Aeromonas caviae* | 0.03 | 0.25 | 2 | 2 | 16 | 32 | 16 | 8 | 32 | ＞64 | ＞8/152 | 4 | 64 | 4 | 128 |
| L7115 | *Aeromonas caviae* | 0.125 | 0.5 | 2 | 1 | 16 | 32 | 16 | 8 | 32 | ＞64 | ＞8/152 | 4 | 64 | 4 | 128 |
| L7125 | *Aeromonas caviae* | 0.125 | 0.5 | 2 | 1 | 16 | 32 | 16 | 8 | 32 | ＞64 | ＞8/152 | 4 | 64 | 4 | 128 |
| L965 | *Aeromonas caviae* | 0.06 | 0.25 | 1 | 0.5 | 2 | 0.25 | 32 | 2 | 4 | 0.25 | ＞8/152 | ＞64 | 4 | 1 | 0.5 |
| L985 | *Aeromonas caviae* | 0.03 | 0.125 | 2 | 0.25 | 2 | 0.25 | 32 | 2 | 4 | 0.25 | ＞8/152 | ＞64 | 4 | 16 | 1 |
| S5112 | *Aeromonas caviae* | 0.03 | 0.25 | 2 | 0.5 | ≤1 | ≤0.008 | ≤0.06 | 2 | ≤0.015 | ≤0.004 | ≤0.5/9.5 | 0.125 | 0.5 | 1 | 0.5 |
| S5162 | *Aeromonas caviae* | 0.06 | 0.25 | 2 | 0.25 | ≤1 | ≤0.008 | 0.125 | 2 | ≤0.015 | ≤0.004 | ≤0.5/9.5 | 0.125 | 0.5 | 1 | 1 |
| S5245 | *Aeromonas caviae* | 0.03 | 0.25 | 2 | 0.125 | 2 | ≤0.008 | 0.125 | 2 | 0.03 | 0.008 | 1/19 | 0.125 | 2 | 1 | 1 |
| S5275 | *Aeromonas caviae* | 0.5 | 0.5 | 2 | 0.25 | 4 | 0.015 | 0.125 | 4 | 0.06 | 0.06 | 1/19 | 0.5 | 4 | 2 | 2 |
| S611 | *Aeromonas caviae* | 0.03 | 0.25 | 0.5 | 0.25 | 4 | 1 | ＞64 | 16 | 32 | 2 | ＞8/152 | ＞64 | 4 | 64 | 8 |
| S621 | *Aeromonas caviae* | 0.03 | 0.25 | 1 | 0.5 | 4 | 1 | ＞64 | 16 | 32 | 2 | 8/152 | ＞64 | 4 | 32 | 16 |
| S8195 | *Aeromonas caviae* | 1 | 0.25 | 2 | 0.125 | ≤1 | ≤0.008 | ≤0.06 | 1 | 0.03 | 0.008 | 1/19 | ≤0.06 | 4 | 1 | 0.5 |
| M621 | *Aeromonas caviae* | 0.03 | 0.125 | 1 | 0.5 | 32 | 1 | 64 | ≤0.5 | 16 | 4 | ＞8/152 | ＞64 | 8 | 64 | 16 |
| M734 | *Aeromonas caviae* | 0.125 | 0.25 | 2 | 1 | 16 | 32 | 16 | 16 | 32 | ＞64 | ＞8/152 | 8 | 64 | 4 | ＞128 |
| M764 | *Aeromonas caviae* | 0.125 | 0.25 | 2 | 2 | 16 | ＞64 | 8 | 16 | 8 | ＞64 | ＞8/152 | 2 | 64 | 4 | ＞128 |
| M975 | *Aeromonas caviae* | 0.06 | 0.25 | 2 | ＜0.125 | 4 | 2 | ≤0.06 | 4 | 0.03 | 1 | 2/38 | 0.25 | 4 | 16 | 1 |
| L924 | *Aeromonas veronii* | 0.25 | 0.5 | 2 | 0.125 | ≤1 | 16 | 0.125 | 16 | 0.06 | 8 | ＞8/152 | 0.25 | 0.25 | 8 | 32 |
| L955 | *Aeromonas veronii* | 0.125 | 4 | 2 | 0.125 | 4 | 16 | ≤0.06 | 16 | 0.06 | 8 | ＞8/152 | 0.25 | 0.25 | 8 | 64 |
| L975 | *Aeromonas veronii* | 0.25 | 0.5 | 1 | 0.125 | ≤1 | 16 | 0.125 | 16 | 0.06 | 32 | ＞8/152 | 0.25 | 0.25 | 8 | 64 |
| L995 | *Aeromonas veronii* | 0.125 | 0.5 | 2 | 0.125 | ≤1 | 16 | 0.125 | 16 | 0.06 | 32 | ＞8/152 | 0.25 | 0.25 | 8 | 64 |
| S5173 | *Aeromonas veronii* | 0.125 | 0.5 | 4 | 0.125 | ≤1 | 0.25 | 0.125 | 8 | 0.06 | 0.125 | 1/19 | 0.125 | 0.25 | 4 | 1 |
| S5183 | *Aeromonas veronii* | 0.125 | 0.25 | 2 | 0.125 | ≤1 | 0.25 | ≤0.06 | 8 | 0.06 | 0.06 | 1/19 | 0.125 | 0.5 | 4 | 1 |
| M194 | *Aeromonas veronii* | 0.06 | 0.25 | 2 | 0.5 | ≤1 | 1 | ≤0.06 | 4 | 0.03 | 0.5 | ＞8/152 | 0.125 | 0.25 | 2 | 32 |
| M662 | *Aeromonas veronii* | 0.06 | 0.25 | 2 | 0.125 | 2 | 2 | ≤0.06 | 4 | 0.06 | 1 | ＞8/152 | 0.5 | 0.125 | ＞128 | 8 |
| M672 | *Aeromonas veronii* | 0.06 | 0.25 | 2 | 0.125 | ≤1 | 1 | ≤0.06 | 4 | 0.06 | 2 | ＞8/152 | 0.5 | 0.125 | ＞128 | 8 |
| M683 | *Aeromonas veronii* | 0.03 | 0.25 | 2 | 0.5 | 4 | 1 | 16 | 8 | 1 | 1 | 4/76 | 64 | 4 | ＞128 | 16 |
| M694 | *Aeromonas veronii* | 0.03 | 0.25 | 2 | 0.5 | 2 | 1 | 16 | 4 | 1 | 1 | 4/76 | 64 | 4 | ＞128 | 16 |

MEM, meropenem; IPM, imipenem; PMB, polymyxin B; TGC, tigecycline; TZP, Piperacillin/tazobactam; LVX, Levofloxacin; ATM, Aztreonam; AMK, amikacin; FEP, Cefepime; CIP, ciprofloxacin; SXT, sulfamethoxazole/trimethoprim; CAZ, ceftazidime; CSL, Cefoperazone/Sulbactam; GEN, gentamycin; TCY, tetracycline.

**Reference**

Borowiak, M., Baumann, B., Fischer, J., Thomas, K., Deneke, C., Hammerl, J.A., et al. (2020). Development of a Novel *mcr-6* to *mcr-9* Multiplex PCR and Assessment of *mcr-1* to *mcr-9* Occurrence in Colistin-Resistant Salmonella enterica Isolates From Environment, Feed, Animals and Food (2011-2018) in Germany. *Front Microbiol* 11**,** 80. doi: 10.3389/fmicb.2020.00080.

Lei, C.W., Zhang, Y., Wang, Y.T., and Wang, H.N. (2020). Detection of Mobile Colistin Resistance Gene *mcr-10.1* in a Conjugative Plasmid from Enterobacter roggenkampii of Chicken Origin in China. *Antimicrob Agents Chemother* 64(10), e01191-20. doi: 10.1128/aac.01191-20.

Parada, A.E., Needham, D.M., and Fuhrman, J.A. (2016). Every base matters: assessing small subunit rRNA primers for marine microbiomes with mock communities, time series and global field samples. *Environ Microbiol* 18(5)**,** 1403-1414. doi: 10.1111/1462-2920.13023.

Walters, W., Hyde, E.R., Berg-Lyons, D., Ackermann, G., Humphrey, G., Parada, A., et al. (2016). Improved Bacterial 16S rRNA Gene (V4 and V4-5) and Fungal Internal Transcribed Spacer Marker Gene Primers for Microbial Community Surveys. *mSystems* 1(1). doi: 10.1128/mSystems.00009-15.
